# Influence of electrohydrodynamics on the drying characteristics and physicochemical properties of garlic

**DOI:** 10.1016/j.fochx.2023.100818

**Published:** 2023-07-31

**Authors:** Bingyang Han, Changjiang Ding, Yun Jia, Huixin Wang, Yuting Bao, Jie Zhang, Shanshan Duan, Zhiqing Song, Hao Chen, Jingli Lu

**Affiliations:** a College of Science, Inner Mongolia University of Technology, Hohhot 010051, China; b Discharge Plasma and Functional Materials Application Laboratory, Inner Mongolia University of Technology, Hohhot 010051, China

**Keywords:** Electrohydrodynamic, Drying, Garlic, Physical and chemical properties, Low-field NMR

## Abstract

Electrohydrodynamic (EHD) drying, natural air drying (AD) and hot air drying (HAD) were used to comprehensively study the drying characteristics and physicochemical properties of garlic, and low-field nuclear magnetic resonance (NMR), infrared spectroscopy, scanning electron microscopy and other technologies were used as detection methods. In terms of drying characteristics, HAD has the largest effective diffusion coefficient of moisture and the shortest average drying time. EHD-treated garlic slices had the most attractive color, the highest rehydration rate, the most stable cell structure, the highest content of active ingredients, and the most stable protein secondary structure. Therefore, electrohydrodynamic drying is a promising garlic slice drying technology and provides an effective method for the large-scale production of high-quality garlic.

## Highlights

• Compared with natural air drying, electrohydrodynamic drying of garlic can improve drying efficiency.
• Compared with hot air drying and natural air drying, electrohydrodynamic drying of garlic has higher quality, such as color, rehydrate, active ingredient content, etc.
• The structure of garlic cells after drying by electrohydrodynamic is more complete.
• Electrohydrodynamic drying provides an effective method for the production of high-quality garlic.

## 1 Introduction

Garlic (*Allium sativum L*.) is used as a functional food as a flavoring agent and herbal medicine around the world because of its protein, polyphenols, and unique allicin (Feng et al., 2019). Epidemiological studies have proven that garlic helps to prevent cardiovascular disease and has anticancer effects and antipathogenic microorganisms because it contains these bioactive components (Feng et al., 2021). In addition, garlic is also a natural antioxidant due to its antioxidant effects (Lau, 2006).

However, the water content of freshly picked garlic exceeds 75%, causing garlic to easily germinate and rot during storage and transportation and thereby producing serious economic losses to growers. Therefore, it is necessary to carry out secondary processing of freshly picked garlic. The results show that the drying process can remove 90% of the water in food, which is an important processing means to delay food decay and reduce the growth of microorganisms (Lamidi, Jiang, Pathare, Wang, & Roskilly, 2019). Therefore, drying is a commonly used secondary processing method for garlic. At present, hot air drying (HAD) is the most commonly used drying method for garlic drying and has been continuously used because of its simple device, simple operation and low capital investment; however, in previous studies, HAD has been found to have shortcomings, such as reduced product quality and nutrient loss (Li et al., 2023, Niu et al., 2020). Therefore, many researchers are seeking a suitable means for drying garlic. Aware and Thorat (2011) studied the effects of many drying methods, including heat pump drying, hot air drying, solar cabinet drying, infrared drying, vacuum drying, freeze drying, and fluidized bed drying, on the drying characteristics, allicin content, and color of garlic flakes. Feng et al. (2021) investigated the effects of vacuum freeze drying, HAD, infrared hot air drying, relative humidity drying, and pulsed vacuum drying on the physical properties, rehydration properties, flavor, bioactive compounds and antioxidant properties of garlic slices.

Electrohydrodynamic (EHD) drying technology is a very promising drying method due to its low energy, low temperature, and other advantages; however, it has not been widely recognized. The dried material has a good effect on color and nutrient retention; thus, the technology of EHD drying is appealing to study (Iranshahi, Rubinetti, Onwude, Psarianos, Schlüter, & Defraeye, 2023). Compared with traditional thermal drying technology, EHD-dried food has much lower degradation in terms of shrinkage and color. In addition, the rehydration capacity of the material after drying in the EHD is also much higher. Elmizadeh et al. (2017) studied the difference between EHD and HAD energy, and the results showed that the energy consumption of HAD was 48.66 times that of EHD. Ni et al. (2020) studied the effects of different pretreatment methods on the drying characteristics and microstructure of goji berries in EHD drying technology. Martynenko and Zheng (2016) studied EHD drying technology for apple flake drying and its effects on drying kinetics and energy consumption. Polat and Izli (2020) applied EHD drying technology to apricot nuggets and showed that EHD could retain the microstructure of the sample better than hot air drying, and no cracks were observed on the surface. In recent years, EHD technology has also been used in the processing of seafood. Bai et al. (2013) applied EHD drying to sea cucumber drying and compared it with natural drying and oven drying, EHD had better performance in sample shrinkage, rehydration rate, protein content and acidic mucopolysaccharides. Tamarit-Pino et al. (2020) applied EHD to the drying of sea cucumbers in Chile and obtained better drying parameters. However, electrohydrodynamic drying technology was not applied to garlic flakes in these studies.

Since HAD is currently the mainstream drying method for garlic (Das & Arora, 2018), in this paper, three methods, HAD, AD and EHD, were used to dry garlic to determine the drying characteristics, effective moisture diffusion coefficient, rehydration rate, surface color difference and allicin potential of garlic flakes under different drying methods and different drying voltages. Scanning electron microscopy and infrared spectroscopy were used to investigate the effects of different drying methods and different drying voltages on the microstructure and chemical composition of the garlic surface. Low-field NMR was performed on completely dried garlic flakes to examine their moisture distribution of the garlic after drying.

## 2 Materials and methods

### 2.1 Original materials

Fresh garlic was purchased from fresh supermarkets near the Inner Mongolia University of Technology (Hohhot, Inner Mongolia). Garlic of the same variety and the same origin with no rot and the same ripeness were selected. The purchased fresh garlic was stored in a cold room at 4 °C for no more than 7 days per experiment. In this experiment, fresh garlic was cut into cylindrical garlic slices with a diameter of 1.2 cm and a thickness of 2 mm using a homemade mold.

### 2.2 Chemical reagents and instruments

The chemical reagents included ultrapure water and a pyruvate detection kit (catalog number: BC2200, specification 50 T/48S).

EHD drying system mainly consisted of a high-voltage power source, a voltage controller (YD (JZ)-1.5/50, Wuhan, China), and a multiple needle-to-plate electrode system. The high-voltage power source could output alternating current (AC) voltage, and it was connected to a voltage controller with an adjustable voltage ranging from 0 to 50 kV for alternating current (AC). The multiple needle electrode is a rectangular disk with a size of 400 mm × 240 mm and 77 needles in total. The length and diameter of each needle were 20 mm and 1 mm, respectively, and the distance between two consecutive needles was 40 mm. The ground electrode was a 1000 mm × 450 mm stainless steel plate.

The temperature range of hot air drying oven (DGX-9053, China) is from 10 °C to 250 °C. The temperature used in this experiment is 50 °C.

### 2.3 Drying experiment

**Scheme 1:**
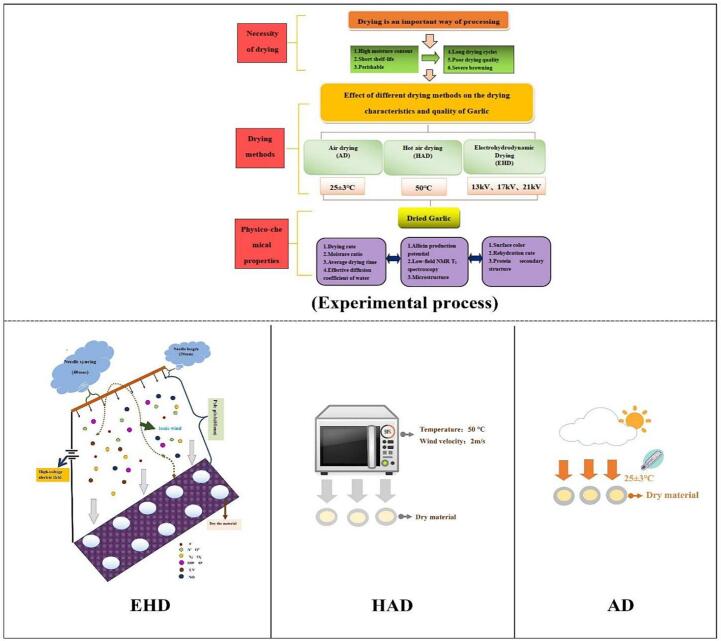
Experimental process and drying model schematic.

In this experiment, AD, HAD, and EHD were used to dry garlic until the moisture content was <10% (dry basis, d.b.). The dried samples were analyzed for drying characteristics, physical properties, rehydration, active ingredient content, low-field NMR, and infrared spectroscopy. An overview of this experiment and a schematic diagram of various drying methods are shown in Scheme 1.

#### Electrohydrodynamic drying

Previous studies have shown that the voltage applied to EHD drying was the most important parameter affecting the quality of dried products. Therefore, fresh garlic flakes were placed in an EHD system that was connected to the ground electrode at voltages of 13, 17, and 21 kV, and the distance from the tip to the material was 60 mm. A schematic diagram of the device is shown in Scheme 1. In the EHD system, the drying temperature, relative humidity and ambient wind speed were 25 ± 2 °C, 27 ± 5% and 0 m/s, respectively. Fresh garlic flakes were placed evenly in the EHD for the drying experiments.

#### Hot air drying

The fresh garlic flakes were placed in an HAD box for drying. The drying temperature was 50 °C, and the drying wind speed was 2 m/s. A schematic diagram of hot air drying is shown in Scheme 1.

#### Natural air drying

The processed garlic flakes were placed in a constant temperature and humidity room with a temperature of 25 °C ± 3 °C and a humidity of 25 ± 5% for drying experiments. A schematic diagram of natural air drying is shown in Scheme 1.

### 2.4 Analysis of the drying characteristics

#### 2.4.1 Determination of the initial water content

The initial water content *M*_0_ of fresh garlic was calculated by placing fresh garlic flakes in an oven at a temperature of 105 °C until the weight was constant (Li et al., 2021). The calculation is shown in Formula (1):

(1) $$M_{0}=\frac{m_{0}-m_{g}}{m_{0}}$$

where *M_0_* is the initial water content of garlic flakes (g/g); *m_0_* is the initial mass of garlic flakes (g); and *m_g_* is the complete mass of dried garlic flakes (g).

#### Determination of the moisture content

The water content and moisture content of garlic flakes during drying are defined as follows (Liu et al., 2021):

(2) $$M_{i}=\frac{m_{i}-m_{g}}{m_{g}}$$

(3) $$\mathrm{MR}=\frac{M_{i}-M_{e}}{M_{0}-M_{e}}$$

where *m_i_* is the mass (g) of garlic flakes when dried to *i* moment, *M_i_* is the water content of garlic flakes when they dry to *i* moment, *MR* is the moisture content of garlic flakes, and *M_e_* is the equilibrium water content of garlic flakes; however, during the drying process of garlic flakes, the equilibrium moisture content was not evaluated, and the *M_e_* value was considered to be zero because the equilibrium moisture content of food raw materials was generally not high (Chauhan, Singh, Dhar, & Powar, 2021). Therefore, the formula for moisture content was simplified to the following:

(4) $$\mathrm{MR}=\frac{M_{i}}{M_{0}}$$

#### Drying rate

The drying rate of garlic flakes is defined as follows:

(5) $$\mathrm{DR}=\frac{{M_{t}-M}_{t+\Delta t}}{\Delta t}$$

where *DR* is the drying rate, *M_t_* is the water content of garlic flakes at time *t*, and *M_t+Δt_* is the water content of garlic flakes at time *t*+Δ*t*.

#### Effective diffusion coefficient of moisture

The effective diffusion coefficient of moisture during the drying process of garlic flakes is calculated using Fick's second law as follows (Ni et al., 2019):

(6) $$\frac{\mathrm{dM}}{\mathrm{dt}}=D_{\mathrm{eff}}\frac{d^{2}M}{dr^{2}}$$

For a long drying process, *MR*<0.6, and the equation can be expressed as follows:

(7) $$\mathrm{MR}=\frac{8}{\pi^{2}}\exp\left(-\frac{\pi^{2}D_{\mathrm{eff}}t}{4L^{2}}\right)$$

where *D_eff_* is the effective diffusion coefficient of the sample and L is half the thickness of the sample. Applying a logarithm on both sides, the following equation is obtained:

(8) $$\ln\left[\;MR\right]=-\frac{\pi^{2}D_{\mathrm{eff}}}{4L^{2}}t+\ln\left[\;\frac{8}{\pi^{2}}\right]$$

*D_eff_* can be derived from the relationship between the ln*[MR]* and time. The slope of the above equation is as follows:

(9) $$\text{k=}-\frac{\pi^{2}D_{\mathrm{eff}}}{4L^{2}}$$

The value of *D_eff_* can be obtained from Equation (9) (Mirzaei-Baktash, Hamdami, Torabi, Fallah-Joshaqani, & Dalvi-Isfahan, 2022).

### 2.5 Physical characteristics analysis

#### Color analysis

The garlic slices before and after drying were directly measured with an automatic colorimeter using the CIE Lab color space, and the brightness value L*, redness value a*, and yellowness value b* on the surface were directly measured. Before testing, the instrument is corrected by blackboard and whiteboard, 5 points are removed from the same sample, and the results are averaged. The expression for chromatic aberration is as follows:

(10) $$\Delta E=\sqrt{{\left(L_{i}^{*}-{\;L}_{0}^{*}\right)}^{2}+{\left(a_{i}^{*}-\;a_{0}^{*}\right)}^{2}+{\left(b_{i}^{*}-{\;b}_{0}^{*}\right)}^{2}}$$

where L_0_*, a_0_*, and b_0_* are the brightness value, redness value and yellowness value of fresh garlic flakes, respectively, and L_i_*, a_i_*, and b_i_* are the brightness, redness and yellowness values of garlic flakes after drying, respectively. The chromatic difference ΔE parameter described in the formula was used to describe the color of the garlic flakes after drying. Chromatic aberration Δ*E* refers to the degree of overall color change of the sample after drying compared to the color of the fresh sample relative to the color value of the fresh sample with color values of L_0_*, a_0_*, and b_0_*.

The whiteness of garlic after drying is also one of the important indicators to evaluate the color of garlic slices, and the whiteness calculation formula can be expressed by Formula (11) (Feng et al., 2021).

(11) $$Whiteness=100-\sqrt{{\left(100-L^{*}\right)}^{2}+{\left(a^{*}\right)}^{2}+{\left(b^{*}\right)}^{2}}$$

where L*, a*, and b* are the brightness value, redness value and yellowness value of garlic flakes before and after drying, respectively.

#### Scanning electron microscopy (SEM)

The dried garlic was attached to the sample stage with carbon conductive glue and gold sprayed, and then the surface structure of the dried garlic flakes was observed at the same position at 5 kV with an accelerated voltage of 5 kV via scanning electron microscopy (SU8020, Japan) at the same magnification of 200 times and 500 times.

#### Rehydration capacity analysis

The dried garlic slices were placed in a beaker, 100 ml of ultrapure water was added, the sample was soaked in a constant temperature water bath at 37 °C until the weight no longer changed, the sample was removed, the excess water on the surface of the garlic slices was absorbed with absorbent paper, and the sample was weighed; the water ratio was calculated according to Formula (12) (Feng et al., 2021):

(12) $$\mathrm{RR}=\frac{W_{g}-W_{0}}{W_{0}}$$

where *W_g_* and *W_0_* are the weights of the sample after rehydration and before rehydration, respectively.

### 2.6 Allicin production capacity analysis

Allicin is produced by the reaction of two alliin molecules catalyzed by alliinase to produce one allicin molecule and two pyruvate molecules (Aware and Thorat, 2011). The pyruvate content can be used to assess the potential of allicin production in garlic, and its level can reflect the strength of allicin's ability. The method for measuring pyruvate was similar to that used by Peng et al. (2019) and Yao et al. (2018), with pyruvate (PA) kits (Solarbio, Beijing, China). The garlic flakes were ground with a mortar until the particles were<200 mesh and mixed with the extract at 1:10; the sample was homogenized in an ice bath for 30 min and then centrifuged at 8000 r/min at room temperature for 10 min with a benchtop centrifuge, and 300 μL of the supernatant were removed. Reagent 1 (100 μL) was mixed with the supernatant; the solution was allowed to stand for 2 min, then 500 μL of Reagent 2 was added and the solution was mixed. The prepared sample was analyzed using a UV spectrophotometer at 520 nm. The pyruvate standard curve was created using standard sodium pyruvate solutions and the absorbance was measured using a spectrophotometer at 520 nm.

### 2.7 Fourier transform infrared spectroscopy (FTIR) analysis

Potassium bromide (130 mg) and 1.3 mg of the sample (the mass ratio of sample to potassium bromide was as high as 1 to 100) were ground into an agate grinding bowl to a particle size that was<2 microns. The powder was pressed into a transparent tablet with a press pressure of 8 tons, and the pellet was then placed into the FTIR for analysis. The scanning range was 400–4000 cm^−1^, with 32 scans at 4 cm^−1^ resolution, and the interference of water and carbon dioxide was removed to obtain the scanning spectrum. The obtained spectral lines were analyzed using PeakFit software (San Rafael, CA, USA) for amide I spectra in the range of 1600–1700 cm^−1^ via baseline adjustment, Gaussian deconvolved, second derivative, and curve fitting.

### 2.8 Low-field nuclear magnetic (LF-NMR) T_2_ spectral analysis

LF-NMR measurement is measured using a nuclear magnetic resonance instrument, the specific LF-NMR test parameters were as follows: temperature 38 °C; main frequency SF of 12 MHz; offset frequency O1 of 556868.43 Hz; 90° pulse time: P90°=5.8 μs; 180° pulse time: P180°=9.84 μs; sampling points: TD=120164; signal reception bandwidth: SW=200 kHz; relaxation decay time: TW=3500 ms; accumulation number: NS=16; echo time: TE=0.15 ms; and echo number: NECH=4000.

### 2.9 Statistical analysis

All experiments were performed 3 times, the final data were averaged, and the standard deviation was obtained by averaging the results of each set of experiments. The results are expressed as the mean ± standard deviation. Differences between groups were determined via one-way analysis of variance (ANOVA). The differences between the means were determined at a significance level of 0.05. The obtained data were normalized, and after processing, Origin software was used to create heatmaps to analyze the correlation between different drying methods and various indicators. In addition, different indicators were analyzed as Pearson correlation analysis to determine the correlation between different indicators.

## 3 Results and analysis

### 3.1 Analysis of drying characteristics

**Fig. 1:**
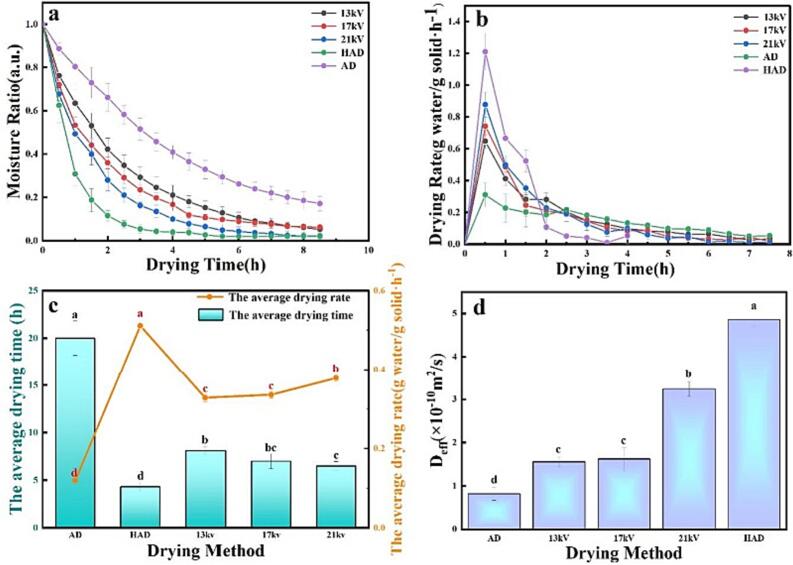
Drying characteristic curve under different drying methods. a: Moisture content over time; b: Drying rate over time; c: Average drying time and average drying rate; d: Effective diffusion coefficient of moisture. Different letters indicate significant differences (*p*<0.05) between sample means.

As Fig. 1a shows, the moisture content decreased at the slowest rate in the AD experimental group and has the constant drying rate. The fastest rate in the HAD experimental group; the decrease rate of moisture content in the EHD experimental group increased with increasing voltage, but the amount gradually decreased with drying time, which was consistent with the results of Xiao and Ding (2022). No constant rate period was observed in the EHD and HAD; hence, the entire drying process occurred in the falling rate period (Fig. 1b). HAD had high temperature, fast gas flow rate, a thin boundary layer between the surface of the dry material and the air, and a high convective heat transfer coefficient. However, the high air flow and convective heat transfer coefficient could lead to rapid dehydration of the garlic surface, resulting in rapid shrinkage at the beginning of drying, causing difficulty for the moisture inside the garlic to spread outward. Therefore, the HAD rate curve showed an initial rapid increase, and then it rapidly decreased. However, the EHD drying rate gradually decreased with the drying progress, as shown in Fig. 1b. EHD drying was predominantly caused by the ion wind generated by the discharge of the needle tip on the surface of the material. Its discharge frequency and current amplitude increase with the increase of voltage. A higher voltage correlated to a larger discharge frequency and current amplitude, a stronger generated ionic wind, and a faster drying speed. Fig. 1c shows the average drying time and average drying rate of different drying methods and different drying voltages, and compared with AD, the average drying times of the EHD drying and HAD were significantly shortened (*p*<0.05), and the average drying rates were significantly improved. Therefore, EHD drying could also be applied to the drying of garlic materials.

#### Effective moisture diffusion coefficient (Deff) analysis

As Fig. 1d shows, the effective moisture diffusion coefficient of the AD method is the lowest, the HAD method had the largest effective water diffusion coefficient, and the effective water diffusion coefficient of EHD increased with increasing voltage. This result was the same as the average drying rate. There was a significant difference in the effective water diffusion coefficient between the different experimental groups (*p*<0.05). Through the effective diffusion coefficient of moisture, hot air drying was more conducive to the diffusion of moisture in the material. EHD had a significantly higher *D_eff_* than AD. EHD was confirmed to be feasible to use for garlic drying.

### 3.2 Surface chromatic aberration analysis

**Fig. 2:**
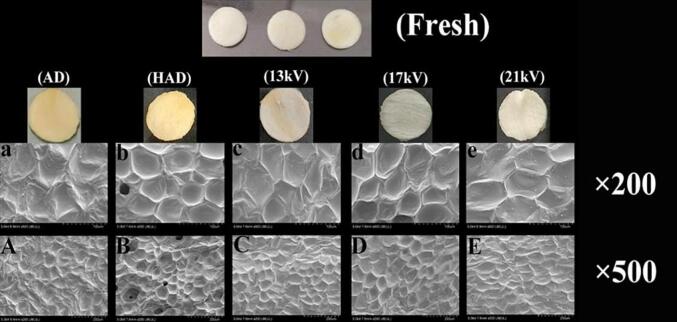
Photos and SEM images of the dried garlic slices treated by different drying techniques. a-e: 200 × magnification; A-E: 500 × magnification; AD: nature air drying; HAD: hot air drying; 13 kV: Voltage of EHD drying is 13 kV; 17 kV: Voltage of EHD drying is 17 kV; and 21 kV: Voltage of field drying is 21 kV.

Color is an important attribute to evaluate the quality of food, affecting consumers' choice and value of products. High-quality dried garlic needs to have higher brightness and whiteness and lower yellowness, specifically, a higher L* and whiteness value correlates to a lower value of a*, b*, and *ΔE*, indicating a higher quality of the dried material (Pei et al., 2021). Table 1 shows the influence of the different drying methods on the surface color of garlic. There is a significant difference in the influence of different drying methods on the surface color of garlic (*p*<0.05), and this difference can be observed in Fig. 2. EHD drying has higher brightness and whiteness, with the highest brightness value of 65.32 ± 1.59 at 21 kV, and the yellowness value of the sample after EHD drying is also significantly lower than those of HAD and AD. This phenomenon occurs because EHD drying and AD are low-temperature drying methods, and the color change in low-temperature drying is caused by enzymatic browning as the main influencing factor (Martynenko and Zheng, 2016). The smaller b* value of EHD relative to AD is potentially due to the rapid evaporation of water due to faster drying speed, which reduces the rate of enzymatic browning and other oxidation reactions of the sample. HAD belongs to hot drying, and the cause of browning of hot dried products is mainly due to the Maillard reaction, caramelization reaction and ascorbic acid browning that occur during the hot drying process (Michalska, Wojdylo, Honke, Ciska, & Andlauer, 2018); thus, HAD has the highest b* value of 22.76 ± 0.15 and the most evident color change value of 8.18 ± 0.48. In summary, EHD drying has unique advantages in preserving the color of garlic compared to AD and HAD. Esehaghbeygi et al. (2014) found that EHD-dried bananas retain their original color well, which is consistent with our findings. The enzymatic browning reactions and color changes in fruits and vegetables are often caused by PPO (polyphenol oxidase) activity, which initiates the oxidation of phenols to quinone. The less color degradation of EHD can be attributed to lower PPO activity, which can also prove that the material after EHD drying has higher antioxidant activity (Iranshahi, Rubinetti, Onwude, Psarianos, Schlüter, & Defraeye, 2023).

**Table 1:** Color change of garlic slices before and after drying under different drying methods.

Voltage	L*	a*	b*	*ΔE*	Whiteness
Fresh	62.97 ± 1.30^b^	0.55 ± 0.095^d^	14.77 ± 0.66^d^	/	60.11 ± 0.96^b^
AD	60.46 ± 0.86^c^	1.69 ± 0.05^b^	20.75 ± 0.86^b^	6.59 ± 0.34^b^	55.29 ± 0.361^d^
HAD	63.28 ± 1.22^b^	2.30 ± 0.17^a^	22.76 ± 0.15^a^	8.18 ± 0.48^a^	56.73 ± 0.77^c^
13 kV	61.80 ± 0.48^bc^	0.70 ± 0.053 ^cd^	17.84 ± 1.92^c^	3.30 ± 1.45^c^	57.79 ± 0.37^c^
17 kV	63.64 ± 0.40^ab^	0.74 ± 0.10^c^	16.23 ± 0.68^d^	1.82 ± 0.30^d^	60.16 ± 0.08^b^
21 kV	65.32 ± 1.59^a^	0.58 ± 0.02^d^	15.82 ± 0.36^d^	2.60 ± 0.14 ^cd^	61.86 ± 1.30^a^

Note: Different letters indicate significant differences (*p*<0.05) between sample means. L*: Brightness value; a*: Redness value; b*: Yellowness value; *ΔE*: Total color difference; Whiteness: Whiteness value.

### 3.3 Microstructure analysis

Fig. 2 shows the physical diagram of garlic after different drying methods and the SEM image; the surface of the material after EHD drying had a more complete cell structure, while the sample surface after HAD appeared to collapse and has defects, and distortion and folds appeared on the surface of the sample after AD. The results were in clear agreement with the rehydration ability of garlic after drying. Based on the SEM image, one of the reasons for the hot air drying results and its fast drying rate was potentially because hot air drying destroyed the surface structure of the material, forming a similar “tunnel” with the inside of the material and resulting in the water inside the material being more easily evaporated. Specifically, the HAD material drying rate was fast, generated under the condition of destroying the surface structure of the material, and is not conducive to the production of high-quality garlic slices. Feng et al. (2021) found the structure of thermally dried garlic samples revealed different degrees of curl, having seriously distorted and deformed cells. Moreover, through observation, the cell structure was more complete after EHD drying, indicating that the bound water content in the garlic cells after drying was higher than those in HAD and AD; this result was also consistent with the conclusion reached by low-field NMR.

### 3.4 Rehydration capacity analysis

**Fig. 3:**
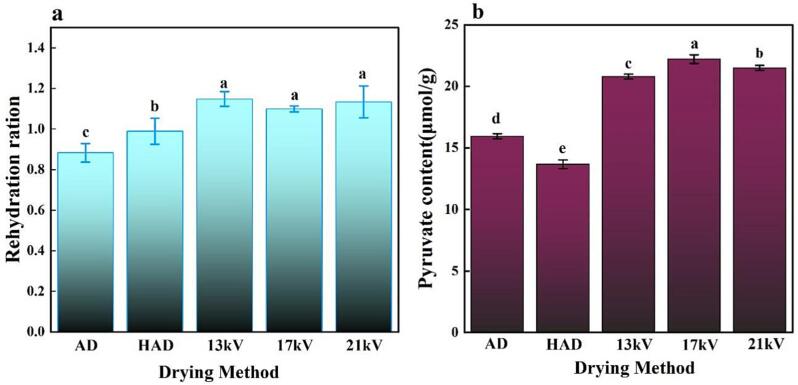
Physical and chemical properties of garlic slices under different drying conditions. a: Effects of the different drying methods on the rehydration capacity of the garlic slices; b: Effect on pyruvate content under different drying methods. Different letters indicate significant differences (*p*<0.05) between sample means.

Rehydration capacity is an important indicator used to evaluate dry products, and the quality of rehydration can be a good indicator of the degree of damage to the material structure (Marques, Prado, & Freire, 2009). A higher rehydration rate corresponds to better product quality, indicating minimal damage to the product structure. Fig. 3a shows that the effect of different drying methods on the rehydration ability of garlic, which is closely related to the change in microstructure and determines the macroscopic properties. From Fig. 3a, the rehydration capacity between different drying methods is significantly different. The AD control group has the worst rehydration capacity; the EHD method is dry, its rehydration capacity is optimal, and there are no evident differences in the rehydration capacity at different voltages (*p*<0.05). This occurs because the EHD drying method is nonthermal drying, and the ionic wind generated only acts on the surface of the material, preventing the possibility of damage to the internal structure of garlic; thus, there is a better rehydration capacity relative to hot air drying. After thermal treatment, the structure of the matrix inside the material collapses, preventing water absorption and reducing the ability to rehydrate. This confirms the conclusion from Liu et al. (2022).

### 3.5 Allicin production potential analysis

The results of measuring pyruvate content are shown in Fig. 3b. The level of pyruvate content reflects the amount of allicin content. The garlic flakes treated by electrohydrodynamic drying had a higher content of allicin than those after natural air drying and HAD; the result was mainly caused by two aspects. The first aspect was relative to HAD at high temperature. High temperature could partially inactivate allinase in garlic, resulting in a relatively low retention content of allicin, while electrohydrodynamic drying was low-temperature drying and more advantageous for retaining the allicin content. This result was similar to the findings of Aware and Thorat (2011). Specifically, low-temperature drying had more advantages in retaining allicin. On the other hand, EHD drying had a faster drying rate than natural air drying, and previous studies found that high-drying rate treatments had higher allicin retention capacity than low-drying rate treatments. Therefore, the effect of the EHD drying method on allicin retention was more significant.

### 3.6 Infrared spectroscopic analysis

**Fig. 4:**
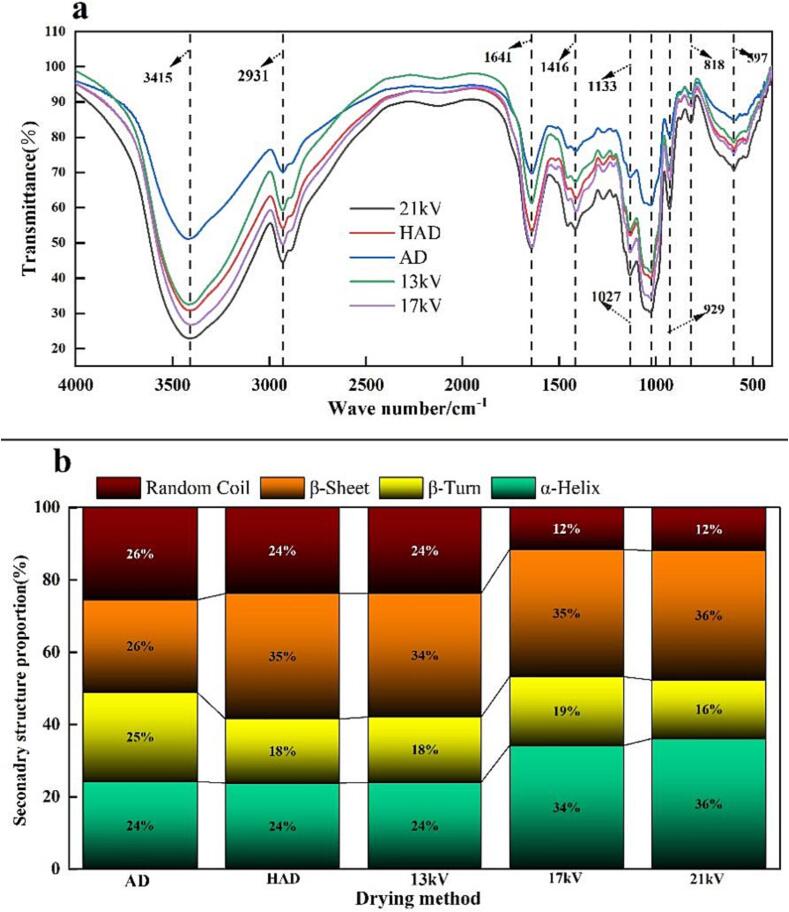
Infrared spectra and the protein secondary structure of garlic slices under different drying conditions. a: Infrared spectra of garlic under different drying methods; b: Proportion of the secondary structure that constitutes the garlic proteins.

Fig. 4 shows the FTIR spectra to determine the effect of different drying methods on the absorption band of garlic flakes; wide and strong tensile vibrations with peaks of N-H and O-H appear at approximately 3415 cm^−1^. The absorption peak at 2931 cm^−1^ corresponds to tensile vibrations, including C-H, such as methyl and methylene groups. Typical protein bands are concentrated at approximately 1640 cm^−1^, 1540 cm^−1^, and 1360 cm^−1^, corresponding to amide I, amide II and amide III groups, respectively. The bands in the region of 900–400 cm^−1^ are mainly attributed to the presence of polysaccharides (Ni et al., 2020). From Fig. 4a, the position of the characteristic peaks produced by different drying methods does not significantly change, indicating that the initial functional group of garlic can be retained regardless of which drying method is used. However, there are evident differences in the strength of the absorption peak, from strong to weak, EHD drying (21 kV) > EHD drying (17 kV) > HAD > EHD drying (13 kV) > AD. Based on the observation of the absorption peak of the protein band, the protein content of garlic after drying at 21 kV and 17 kV is higher than that of HAD. This result shows that drying with an electric field at a suitable voltage is more beneficial than HAD and is more conducive to retaining the nutrients in garlic.

The protein content in garlic accounts for a large proportion, protein is a large molecule, and the secondary structure directly determines its properties; thus, it is particularly important to explore secondary structure of proteins in garlic tablets. The resulting transmission lines were analyzed for the amide I spectra in the range of 1600–1700 cm^−1^ via baseline adjustment, Gaussian deconvolution, second derivative, and curve fitting. The peak position is used to quantify changes in the proportion of the protein secondary structures: α-Helix at 1646–1664 cm^−1^; β-Sheet at 1615–1637 cm^−1^ and 1680–1700 cm^−1^; β-Turn at 1664–1680 cm^−1^; and random coil at 1637–1645 cm^−1^. The results obtained are shown in Fig. 4b, α-helix and β-sheet are the main forms of the secondary structure of proteins in garlic tablets. The content of α-helix after 21 kV treatment was not significantly different from that in the 17 kV group, and the content of α-helix was higher than those of HAD and AD, indicating that the protein secondary structure stability of garlic slices under EHD treatment was higher under a suitable voltage. In addition, the protein disordered structure of garlic slices treated with HAD, AD and 13 kV treatment increased, indicating that the protein secondary structure of garlic slices treated by these means was damaged. This is potentially due to the high temperatures as well as prolonged drying, resulting in formation. Equivalent results have been found in previous studies. In general, EHD drying has more evident advantages in retaining the nutrient content of the material.

### 3.7 Low-field NMR analysis

**Fig. 5:**
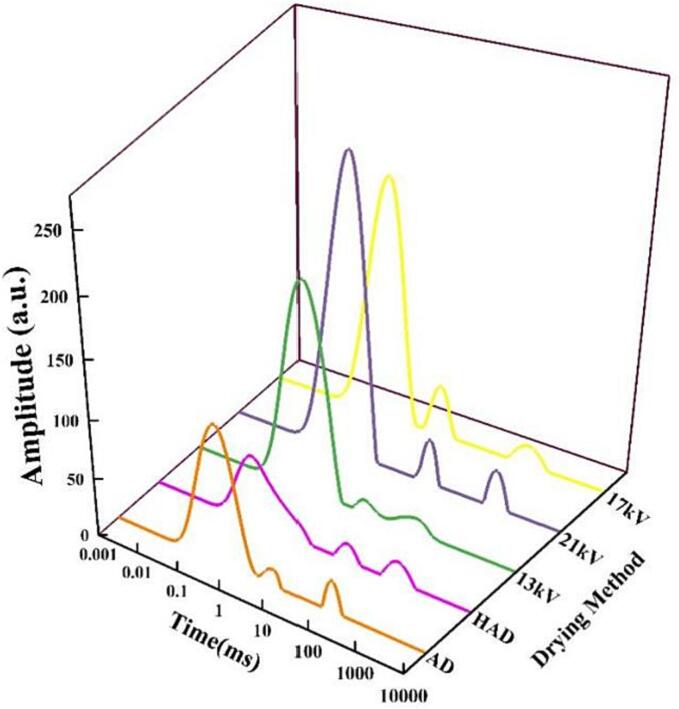
Lateral relaxation time (T_2_) spectrum of the garlic flakes after drying under different drying conditions.

Moisture content and moisture activity are key indicators to ensure the safety and quality of dry products (Jia, Wang, Li, & Liu, 2016). To produce high-quality food, it is crucial to understand the changes that occur in moisture during the drying process. LF-NMR has the advantage of noninvasive quantitative analysis and is widely used in the detection of moisture status and distribution in food raw materials (Chen et al., 2020, Cheng et al., 2017, Geng et al., 2015; M. Li et al., 2014). In previous studies, most researchers have used low-field NMR techniques to detect the distribution of water during garlic drying. For samples after drying, the moisture status of the sample is less studied. Therefore, in our study, garlic is dried until the quality does not significantly change, and the sample is analyzed via low-field NMR. The T_2_ line is shown in Fig. 5. As in previous studies, the dried garlic exhibited three peaks. Peak T_21_ has the shortest relaxation time (0.1–10 ms) and represents the bound water closely related to macromolecular particles. The relaxation time of peak T_22_ (10–80 ms) represents the fixed water trapped in the cytoplasm. Peak T_23_ has the longest relaxation time (80–600 ms) and represents the free water in the vacuole and intercellular space (Chen et al., 2020). From Fig. 5, after drying, the free water content of the material corresponding to each drying method disappears, indicating that the drying method predominantly focuses on the free water in the material. However, interestingly, the content of the bound water after different drying conditions is different. The bound water content of the material dried by EHD is significantly higher than those of HAD and AD, and with increasing voltage, the content of the bound water increases. The combined water content of the material after HAD is the lowest because the high temperature potentially promotes the cellular metabolic process, and the content of free water restricts the metabolic activity of the cell; thus, in the HAD process, part of the bound water of the garlic slices is converted into free water, and the free water is evaporated. The content of bound water in garlic flakes after drying by the electric field is the highest because the high-energy particles produced by the EHD drying ionized air potentially acts on the water molecules in the garlic flakes, increasing the polarity of the water molecules in the garlic flakes, and the binding ability of the amino groups, hydroxyl, carboxyl groups and other substances in the material is enhanced. This binding capacity increases with increasing voltage, promoting the formation of bound water. This also confirms that drying technology changes the proportion of free hydrogen ions in the material and that EHD drying only acts on the surface of the material, producing a more complete cellular structure of the material. Bound water is an important part of the cell structure. HAD and high temperature destroy the structure of the cells, resulting in a lower content of bound water. The SEM results can also show that HAD destroys the structure of the material. Therefore, EHD drying has evident advantages in maintaining the cell structure.

### 3.8 Statistical analysis

**Fig. 6:**
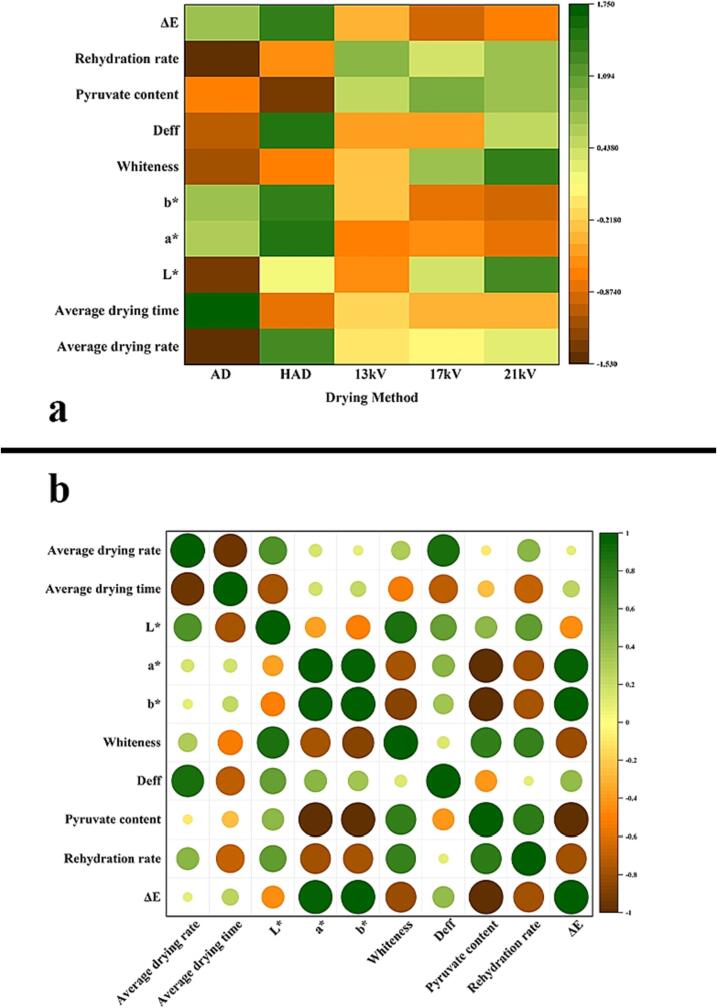
Statistical analysis. a: Correlation heatmap of the drying method and drying index; b: Pearson correlation coefficient matrix.

The obtained experimental data (average drying rate, average drying time, effective moisture diffusion coefficient, rehydration rate, pyruvate content, and chromaticity value) were normalized, and correlation analysis was carried out. Fig. 6a shows the correlation of the effect of different drying methods on the drying index of garlic flakes. HAD is better than EHD drying in terms of drying characteristics; however, the other indicators show that electric field drying has more advantages, among which L* and whiteness have the largest correlation coefficient at 21 kV, and a* and b* have the largest negative correlation coefficient. The results show that the color of garlic flakes after drying at 21 kV is more attractive. At a voltage of 13 kV, the hydration ability showed the largest correlation coefficient, indicating that the rehydration ability of garlic flakes was optimal at 13 kV, the cell structure was more stable, and the pyruvate content had the largest correlation coefficient at a voltage of 17 kV, indicating that at a voltage of 17 kV, there was greater retention of garlic. Fig. 6b is a matrix of Pearson's correlation coefficients, which shows the correlation between different indicators; the drying rate is positively correlated with the color value (L*, b*, a*, and whiteness), the effective diffusion coefficient of moisture, and the ability to replenish water, indicating that the drying speed affects the color of the material after drying, and a faster drying speed correlates to a more complete color retention of the material. Notably, the redness value a*, yellowness value b* and color difference *ΔE* value have an evident negative correlation with the pyruvate content and rehydration ability. The color value of the dried sample can indirectly reflect the content of active ingredients and the damage inside the cells after drying, providing a faster method for evaluating drying technology.

## 4 Conclusion

Based on the above research and discussion, different drying technologies have significant differences in the drying characteristics of materials, the physical properties of dried materials and the quality of materials. Through analysis, the drying characteristics of hot air drying, including drying rate, average drying time, and effective diffusion coefficient of moisture, are better than those of EHD drying. However, in terms of the quality of the material and the retention of the active ingredients (including the surface structure, surface color difference, rehydration ability, and protein secondary structure of the material after drying), large-field drying is significantly better than that of traditional HAD. In conclusion, our results provide a new drying technique for garlic drying and theoretical support for garlic drying with the EHD drying technique.

## CRediT authorship contribution statement

**Bingyang Han:** Conceptualization, Investigation, Methodology, Writing – original draft. **Changjiang Ding:** Conceptualization, Project administration, Resources, Supervision, Writing – review & editing. **Yun Jia:** Data curation, Visualization, Validation. **Huixin Wang:** Data curation, Visualization, Validation. **Yuting Bao:** Data curation, Visualization, Validation. **Jie Zhang:** Data curation, Visualization, Validation. **Shanshan Duan:** Data curation, Visualization, Validation. **Zhiqing Song:** Conceptualization, Methodology. **Hao Chen:** Conceptualization, Methodology. **Jingli Lu:** Conceptualization, Project administration, Resources, Supervision, Writing – review & editing.

## Declaration of Competing Interest

The authors declare that they have no known competing financial interests or personal relationships that could have appeared to influence the work reported in this paper.

## Data Availability

Data will be made available on request.
